# Catechol-Modified Alkali Lignin for Cr (VI) Removal from Synthetic Wastewater

**DOI:** 10.3390/polym17121658

**Published:** 2025-06-15

**Authors:** Chenkun Yu, Ze Liang, Ruoyao Zhou, Tingting Gao, Zhaojiang Wang, Xiaoxia Cai, Qian Lu, Cong Li, Jinshui Yao, Qinze Liu

**Affiliations:** 1 School of Materials Science and Engineering, Qilu University of Technology (Shandong Academy of Sciences), #3501 Daxue Road, Western University Science Park, Jinan 250353, China; 19861651825@163.com (C.Y.); 15634109785@163.com (Z.L.); 13173004716@163.com (R.Z.); qluqlu@qlu.edu.cn (Q.L.); yaojsh@qlu.edu.cn (J.Y.); 2 School of Chemistry and Chemical Engineering, Qilu University of Technology (Shandong Academy of Sciences), Jinan 250353, China; ttgao@qlu.edu.cn; 3 School of Key Laboratory of Paper Science & Technology of Ministry of Education, Qilu University of Technology (Shandong Academy of Sciences), Jinan 250353, China; wzj820415@126.com; 4 State Key Laboratory of Biobased Material and Green Papermaking, Qilu University of Technology (Shandong Academy of Sciences), Jinan 250353, China; congli2016@163.com

**Keywords:** lignin, chromium (VI), chromium (III), adsorption

## Abstract

Chromium (III) ions are essential for biological functions, whereas chromium (VI) ions (Cr (VI)) pose toxicity risks to both humans and animals. Therefore, it is crucial to remove these ions from industrial sources. In this work, to remove hazardous Cr (VI) from wastewater or convert it to Cr (III), catechol-modified alkali lignin (CAL) was prepared using catechol, acetone, and alkali lignin, which is a byproduct in the paper-pulping process. The sample was characterized using a combination of techniques, including scanning electron microscopy, thermogravimetric analysis, Fourier transform infrared spectroscopy, and X-ray photoelectron spectroscopy. Various factors influencing the adsorption behavior of CAL were investigated. The adsorption behavior aligns with the pseudo-second-order kinetic model and adheres to the Langmuir isotherm model. CAL simultaneously achieves Cr (VI) adsorption (498.4 mg/g) and reduction (54.6% to Cr (III)), surpassing single-function lignin adsorbents by integrating catechol’s redox capacity with lignin’s structural stability, which is another way to efficiently utilize Cr (VI) solutions. The mechanism of adsorption and reduction is discussed, which is influenced by its functional groups. In brief, this method paves a new path for the utilization of alkali lignin and provides novel opportunities for the removal of Cr (VI) contamination.

## 1. Introductory

Chromite is hard, wear-resistant, heat-resistant, and corrosion-resistant, and it is extensively applied in the field of metallurgy and refractory materials and in chemical and other industries [1,2]. However, according to [3,4,5], Cr (VI), which is generated during these industrial production activities and discharged into the environment with wastewater, can be harmful to human health, resulting in issues such as lung complications and liver and kidney disease, and it may also cause cancer [6,7,8]. Because of the permeability and biotransformation characteristics of Cr (VI), it not only affects human beings but also the growth and development of bacteria, animals, and plants [9,10]. Yet, Cr (III) can regulate blood sugar and insulin levels, protect the liver, and also improve moods [11]. Therefore, it is imperative to address the treatment of Cr (VI) in wastewater and convert it into beneficial Cr (III) as much as possible so as to realize the efficient use of chromite resources [12].

So far, different methods have been applied to decrease Cr (VI) water pollution, such as chemical precipitation [13], membrane separation [14], ion exchange [15], photocatalytic oxidation, adsorption, and other synergistic purification methods [16]. Among the aforementioned methods, adsorption is an economical, simple, and easy-to-use method with high efficiency in removing water pollutants [17,18]. Different kinds of adsorption materials are utilized, including bio-based organic, synthetic–organic, inorganic, and composite materials [19]. Compared to others, bio-based materials have the advantages of being environmentally friendly, having a wide source of raw materials, being easy to operate, and being safe [20,21]. Thus, they are attracting more and more attention and have a broad development prospect in wastewater treatment.

Catechol is a strong reducing agent that can reduce Cr (VI) to Cr (III) [22]. It is soluble and not suitable for direct use in the reduction and removal of Cr (VI) in wastewater [23]. Lignin is the second-largest biological resource and a complex natural organic aromatic polymer in plants [24,25,26]. It possesses the advantages of being environmentally friendly, renewable, and widely available [27,28]. Alkali lignin, a major byproduct of the kraft pulping process, constitutes 30–45% of black liquor solids. Its amphiphilic structure contains three types of phenolic subunits (p-hydroxyphenyl, guaiacyl, and syringyl) linked by β-O-4 bonds, providing abundant sites for chemical modification [29]. Annually, >50 million tons is generated globally, yet <5% are valorized, with most being incinerated for low-value energy recovery [30,31]. The adsorption capacity of alkali lignin for heavy metals primarily arises from its phenolic -OH groups that form complexes with metal ions and electron-rich aromatic rings, facilitating π–cation interactions and sulfonate groups in sulfonated derivatives that strengthen ionic binding [32]. Nevertheless, unmodified alkali lignin exhibits constrained adsorption capacities due to its modest surface area and inadequate redox-active functional groups [33].

Before presenting our approach, it is important to clarify the unresolved challenges in this field. In this study, catechol modification was specifically chosen over alternative functionalization methods (e.g., sulfonation, amination) for several key reasons. The primary advantage lies in the phenolic hydroxyl groups of catechol, which provide both adsorption sites and electron-donating capacities for Cr (VI) reduction, enabling dual functionality that is unattainable through sulfonation or amination alone. Furthermore, catechol’s aromatic structure enhances π–π interactions with lignin’s backbone, conferring superior stability compared to aliphatic modifications. Equally important, the reaction conditions for catechol grafting (acid-catalyzed phenolization) are more environmentally benign than sulfonation (requiring concentrated acids) or amination (often needing toxic reagents). This approach builds on the recent work by Zhang et al. [34] while uniquely adapting it to alkali lignin for enhanced cost-effectiveness.

In this article, in order to enhance the adsorption of alkali lignin, alkali lignin was phenolically modified with the more reactive catechol (CA) to obtain high-performance adsorbent materials (CAL). This modification improved the adsorption capacity of alkali lignin while retaining the reduction capacity of catechol. The synthesized CAL was characterized, and an investigation was conducted with respect to the impact of various factors on the removal efficiency of Cr (VI). In addition, reduction was also explored.

## 2. Materials and Methods

### 2.1. Material

Alkali lignin was purchased from Hunan, China Juntai New Material Technology Co. Catechol (AR) and potassium dichromate (99.8%) were obtained from Aladdin (Shanghai, China). Acetone (AR), Sulfuric Acid (AR), and Hydrochloric Acid (AR) were purchased from Yantai, China Far East Fine Chemical Co. Sodium hydroxide (AR) was purchased from China Sinopharm Chemical Reagent Co. All experiments were performed using deionized water (UW).

### 2.2. Preparation of CAL

**Figure 1:**
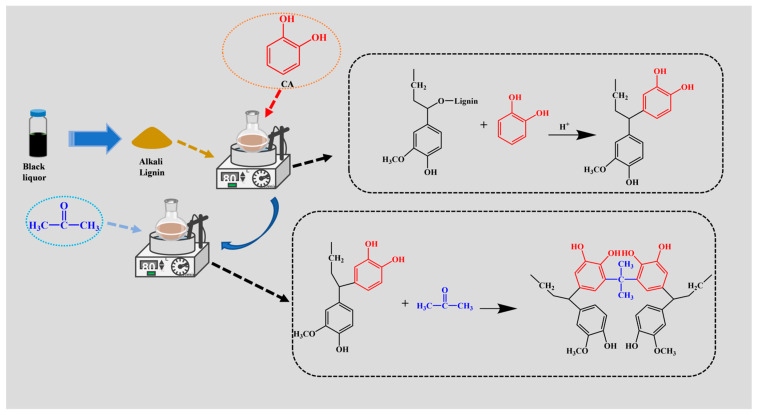
Schematic diagram of the preparation process of CAL (black liquor: industrial byproduct containing raw alkali lignin).

A mixture of alkali lignin (3.0 g, ~18 mmol phenylpropane units, assuming Mw ~180 g/mol) and catechol (2.0 g, 18.2 mmol) at a 1:1 molar ratio (relative to phenylpropane units) was mixed well and placed in a 200 mL three-necked flask. H_2_SO_4_ (72%, 4 mL) was slowly added dropwise to the mixture, and the reaction was heated at 80 °C under a nitrogen atmosphere with continuous stirring for 2 h. Subsequently, an adequate amount of acetone was gradually added in drops to the reaction system and allowed to react for 8 h. After the reaction, we dialyzed the mucilage for 48–72 h until the dialysate was neutral. After centrifugation and freeze-drying for 24 h, the catechol-modified alkaline lignin (CAL) was obtained. The preparation process and its possible reaction mechanism are shown in Figure 1.

### 2.3. Physicochemical Characteristics

The micromorphology of the adsorbent was examined using a scanning electron microscope (FEI QUANTA FEG250 Thermal Field Emission Spectroscopy ,Milan, Italy: Oxford INCA X-MAX50). This advanced configuration enabled nanoscale resolution (<5 nm) for the precise evaluation of particle size distribution, surface roughness, and pore structure, all critical for understanding adsorption behavior. Fourier transform infrared spectroscopy (FT-IR, NICOLET iS10, Massachusetts, America) was employed to characterize infrared absorption groups across 4000–400 cm⁻¹ using the potassium bromide pellet method. The heat loss properties of the materials were characterized by a thermogravimetric analyzer (TGA, Mettler Toledo, Greifensee, Switzerland) at a flow rate of 10 °C min^−1^ under a nitrogen atmosphere. X-ray photoelectron spectroscopy (XPS, Thermo SCIENTIFIC ESCALAB 250Xi, Massachusetts, America, was used to document changes in the surface composition of the material before and after adsorption. A UV-Vis spectrophotometer (UV-2550, Shimadzu, Japan) and an Inductively Coupled Plasma Optical Emission Spectrometer (ICP, Agilent 5800 ICP-OES, California, America) were utilized to quantify the concentrations of Cr (VI) and Cr (III), both before and after the adsorption process. The pH of the Cr (VI) solution before adsorption was adjusted with a pH meter (PHS-25, Shanghai, China). The adsorption experiments were carried out in a water-bath thermostatic shaker (SHA-CA, Changzhou, China).

### 2.4. Batch Adsorption Experiments

The stock solution of Cr (VI), with a concentration of 2000 mg/L, was prepared by dissolving an appropriate amount of K_2_Cr_2_O_7_ powder in UW, which was stored in a 1000 mL volumetric flask. Other Cr (VI) solutions of various concentrations were prepared through the dilution of the aforementioned reserve solution. The pH values were adjusted by using a 1 mol L^−1^ solution of NaOH and HCl. The adsorption experiments were conducted by introducing a specific mass of CAL into a 250 mL conical flask, which contained 100 mL of Cr (VI) solution. The flask was transferred to a water-bath thermostatic shaker running at a pre-set temperature and 200 rpm. At the conclusion of the adsorption process, the supernatant was obtained from the mixture solution through filtration and centrifugation. The actual concentration of Cr (VI) and Cr (III) was calculated by UV spectrophotometry (540 nm using the 1,5-diphenylcarbazide method. Detection limit: 0.01 mg/L, R^2^ > 0.999 for 0–10 mg/L calibration) and its combination with ICP (detection limit: 0.001 mg/L, with Cr (III) calculated by difference) emission spectrometry [35,36]. The initial pH (2–7), water bath temperature (20, 30, and 40 °C), contact time (0–48 h), initial Cr (VI) concentration (0–250 mg L^−1^), and adsorbent dosage (15–45 mg) were systematically evaluated to examine the performance characteristics of the adsorbents. All experiments aimed at removing Cr (VI) were conducted in duplicate to verify the authenticity of the collected data.

The adsorption capacity and removal of Cr (VI) by CAL can be calculated by the following equation:

(1) $$Q_{e}=\frac{\left(C_{0}-C_{e}\right)V}{m}$$

(2) $$Q_{t}=\frac{\left(C_{0}-C_{t}\right)V}{m}$$

(3) $$R\%=\frac{\left(C_{0}-C_{t}\right)}{C_{0}}\times 100\%$$

where *C*_0_ (mg L^−1^) denotes the initial dye concentration, *C_t_* (mg L^−1^) denotes the remaining concentration at a particular moment, *C_e_* (mg L^−1^) denotes the concentration at equilibrium, *Q_t_* (mg g^−1^) denotes the capacity of adsorption at any moment, *Q_e_* (mg g^−1^) denotes the capacity of adsorption at equilibrium, *V* (mL) is the volume of Cr (VI) solution, and *m* (g) is the mass of CAL.

## 3. Results and Discussion

### 3.1. Characterization of the CAL Structure

**Figure 2:**
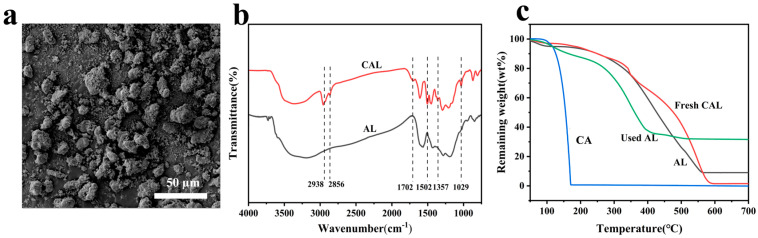
(**a**) Schematic diagram of the preparation process of CAL; (**b**) FT-IR spectroscopy of AL and CAL; (**c**) TG curves of AL, CA, and CAL before and after Cr (VI) adsorption.

SEM revealed CAL particles with irregular morphology and a 5–25 µm size distribution (Figure 2a). These particles were composed of smaller subunits, resulting in their rough surface. The particle size was about 10 µm. The smaller particle sizes with more uniform dispersion of surface-attached particles demonstrated greater structural stability, which enhanced Cr(VI) adsorption on the particle surfaces.

Figure 2b presents the FT-IR spectra of AL and CAL. Comparing the spectra of AL, the adsorption peaks at 2938 and 2856 cm ^−1^ in CAL, which are attributed to C-H stretching vibrations [34], appear significantly enhanced. This indicated that the acetone successfully reacts with catechol and lignin. In addition, the peak at 1357 cm^−1^ also confirms this. The peak at 1720 cm^−1^ is due to C = O stretching, which is attributed to the fact that some phenolic group is oxidized [37]. The absorption peak at 1029 cm^−1^ is caused by the C-O vibrations of the aliphatic ether bond on lignin [38]. A new absorption peak at 1506 cm^−1^ is observed in the CAL spectra compared to the AL spectra. It is attributed to the aromatic ring vibration of catechol, which is a good indication of the successful introduction of catechol [39].

The TG curves of AL, AL, and CAL before and after Cr (VI) adsorption are shown in Figure 2c. Catechol (CA), being a small molecule, lost its weight nearly completely. AL exhibited its weight mainly in two stages. The first stage was before 150 °C, which was due to the loss of water in lignin [40]. The second stage was after 200 °C, which was due to the decomposition of lignin [41,42]. There was about 10% residual, which was attributed to the inorganic ions in AL. The thermal stability of CAL was similar to that of AL. Yet the weight loss of CAL was less than that of AL, and the residual of CAL was less than that of AL, which can be attributed to the cleaning of the inorganic ions in the process of preparing CAL. In the range of 200–350 °C, CAL was more stable than AL, which can be attributed to cross-linking [43]. The CAL after Cr (VI) absorption (the used CAL in Figure 2c) was less stable than the CAL before. This can be attributed to the fact that the presence of chromium can destroy the intermolecular interactions between CAL or enhance the thermal conductivity of the adsorbent. The residual of CAL after Cr (VI) adsorption is about 40%, and this can be attributed to the loading or chromium.

### 3.2. Effect of the Monomer and Ratio Composition on the Adsorption Amount

**Figure 3:**
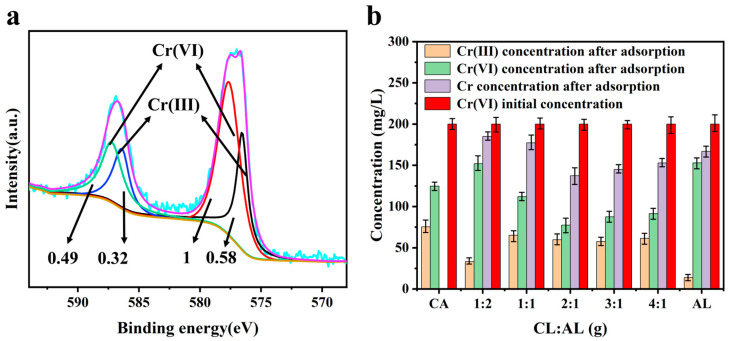
(**a**) XPS analysis after the CA reaction with chromium (pH = 2, C_0_ = 200 mg L^−1^, V = 100 mL, CA dosage = 25 mg, t = 24 h). (**b**) Effect of AL, CA, and CA/AL mass ratio on the adsorption performance of the adsorbent.

Catechol (CA) acts as a reducing agent and is soluble in water. The solid obtained after the evaporation of the mixed solution of CA reacted with Cr (VI) was analyzed by XPS, and about 37.66% of Cr (VI) was reduced to Cr (III), as shown in Figure 3a. Cr (VI) was reduced by CA to Cr (III). The reduction amount was 301.28 mg g^−1^ (Figure 3b). This indicated that the oxidized catechol and Cr (III) could not form sediment in this situation. AL can also adsorb and reduce Cr (VI). The values were 132.4 mg g^−1^ and 55.6 mg g^−1^, respectively. This indicated that AL and chromium can form sediment, and AL can reduce Cr (VI) ions, which confirms the role of the phenolic hydroxyl group.

The effect of different feedstock ratios (CA: AL = 1:2, 1:1, 2:1, 3:1, 4:1) on Cr (VI) removal is shown in Figure 3. Compared to that of AL, the residual concentrations of Cr (III) increased to 33.1 mg L^−1^, 65.2 mg L^−1^, 59.8 mg L^−1^, 57.5 mg L^−1^, and 61.4 mg L^−1^ for CAL. This indicated that the introduction of CA can improve the reduction property. Compared to that of AL, the adsorption and reduction amounts of CAL (2:1) were higher: the value reached 260.4 mg g^−1^ and 238.0 mg g^−1^. While the CA/AL ratio reached 1:1 or 3:1, the adsorption amount decreased. Therefore, the 2:1 CA:AL ratio was selected for subsequent experiments (Table S1).

### 3.3. Effect of Time

**Figure 4:**
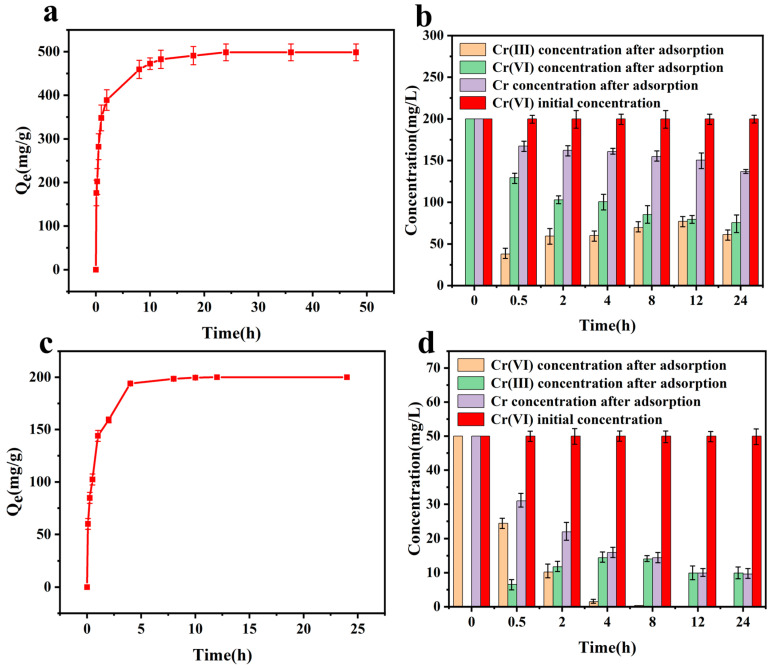
(**a**,**b**) Contact time (pH = 2, C_0_ = 200 mg L^−1^, CAL dosage = 25 mg, V = 100 mL, T = 30 °C, t = 0~48 h). (**c**,**d**) Contact time (pH = 2, C_0_ = 50 mg L^−1^, CAL dosage = 25 mg, V = 100 mL, T = 30 °C, t = 0~24 h).

Contact time is a crucial factor in the adsorption process, as it reflects the attainment of adsorption equilibrium. The amount of adsorbed and reduced Cr (VI) (0–48 h), following the time, is shown in Figure 4. It can be found that the equilibrium time was 24 h, and the total amount was about 500 mg g^−1^. In addition, it is interesting to note that about an amount of 80% was reached within the first 4 h (Figure 4a). This indicated that CAL can quickly adsorb and reduce Cr (VI). The concentrations of each component in the solution are shown in Figure 4b. The residual Cr (VI) decreased slowly as the contact time increased. When the time ranged from 12 h to 24 h, the concentration of Cr (VI) did not change obviously. For the concentration of Cr (III), the value increased in the first 12 h and decreased in the next. This may indicate that CAL can reduce Cr (VI) first and then adsorb the formed Cr (III).

When the initial concentration of the Cr (VI) solution was reduced to 50 mg/L and the other conditions remained unchanged, the amount of reduced adsorbed Cr (VI) could be achieved in 12 h (Figure 4c,d). Consistent with previous observations, the residual concentration of Cr (VI) decreased gradually, while the concentration of Cr (III) increased first and then decreased. Yet Cr (VI) could reach less than 0.01 mg L^−1^. Compared to the concentration at 12 h and 24 h, Cr (VI) did not increase. This interesting result may indicate that Cr (III) cannot be easily oxidized by O_2_ in air in this situation.

### 3.4. Effect of Initial Target Ion Concentration and Temperature

**Figure 5:**
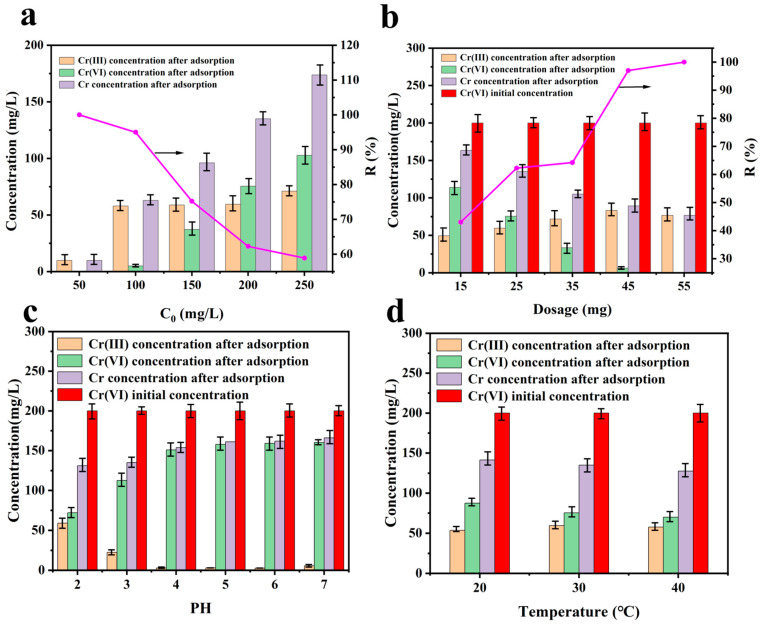
(**a**) Initial Cr(VI) concentration (pH = 2, C_0_ = 50~250 mg L^−1^, CAL dosage = 25 mg, V = 100 mL, T = 30 °C, t = 24 h); (**b**) CAL dosage (pH = 2, C_0_ = 200 mg L^−1^, CAL dosage = 15~55 mg, V = 100 mL, T = 30 °C, t = 24 h); (**c**) pH (pH = 2~7, C_0_ = 200 mg L^−1^, CAL dosage = 25 mg, V = 100 mL, T = 30 °C, t = 24 h); (**d**) temperature (pH = 2, C_0_ = 200 mg L^−1^, CAL dosage = 25 mg, V = 100 mL, T = 20, 30, 40 °C, t = 24 h) on Cr (VI) adsorption by CAL.

The effects of the initial concentration of Cr (VI) and dosage of CAL on the removal and reduction of Cr (VI) are shown in Figure 5a,b. When the concentration was lower (e.g., less than 50 mg L^−1^), or the dosage was higher (e.g., more than 55 mg), the residual concentration of Cr (VI) could be decreased to nearly zero, while the Cr (III) kept a certain concentration. The decrease may be attributed to the ratio of CAL/Cr(VI), which affects the reduction and adsorption. The maintained Cr (III) concentration can be attributed to the poor adsorption property of the phenolic hydroxyl group and the limited adsorption property of the o-benzoquinone group toward Cr (III).

With the increase in temperature, the adsorption and reduction amount appeared to rise, as shown in Figure 5d. On the one hand, increasing temperature is favorable for the continuous exposure of active sites on CAL, benefiting adsorption and reduction. On the other hand, it promotes the migration of ions in the solution, helping to overcome the energy barrier.

### 3.5. Effect of pH

The pH of the solution affects the solution chemistry and the surface functional group state of the adsorbent, which is an important environmental factor controlling the performance of the adsorbent for the adsorption of pollutant ions. As shown in Figure 5c, the residual concentration of Cr (VI) in the solution increased as the pH values increased. The pH-dependent performance of CAL arises from synergistic chemical effects. Under acidic conditions (pH < 4), HCrO_4_^−^ predominates and readily binds to protonated catechol (−OH_2_^+^), while alkaline conditions (pH > 6) favor CrO_4_^2−^ formation, which is repelled by deprotonated CAL (−O^−^). Additionally, OH^−^ competition at pH > 5 reduced Cr(VI) uptake by ~75% compared to the optimal acidic conditions (Figure 5c inset), explaining the peak performance at pH 2–4. And these values did not change obviously as the pH value increased to 5–7. This means that the amount removed decreased quickly when the pH value was less than 4 and slowly when the pH value was more than 4. This is similar to that reported before [44]. Yet, the Cr (III) concentration decreased before pH 4 and increased a little after 4. This indicated that the CAL reduction decreased as the pH value increased and maintained a certain reducing property in weakly acidic conditions. When the pH was 7, there was a slight increase in the concentration of Cr (III). This can be attributed to the reduction of phenolic hydroxyl groups, which involves an electron transfer process. During this process, phenolic hydroxyl groups are converted into phenoxyl radicals. These radicals act as electron donors, reducing Cr (VI) to Cr (III) [45]. Under near-neutral conditions, phenolic hydroxyl groups predominantly exist in the form of phenoxyl radicals and exhibit a stronger tendency to donate electrons for the reduction of Cr (VI) [46].

### 3.6. XPS Analysis

**Figure 6:**
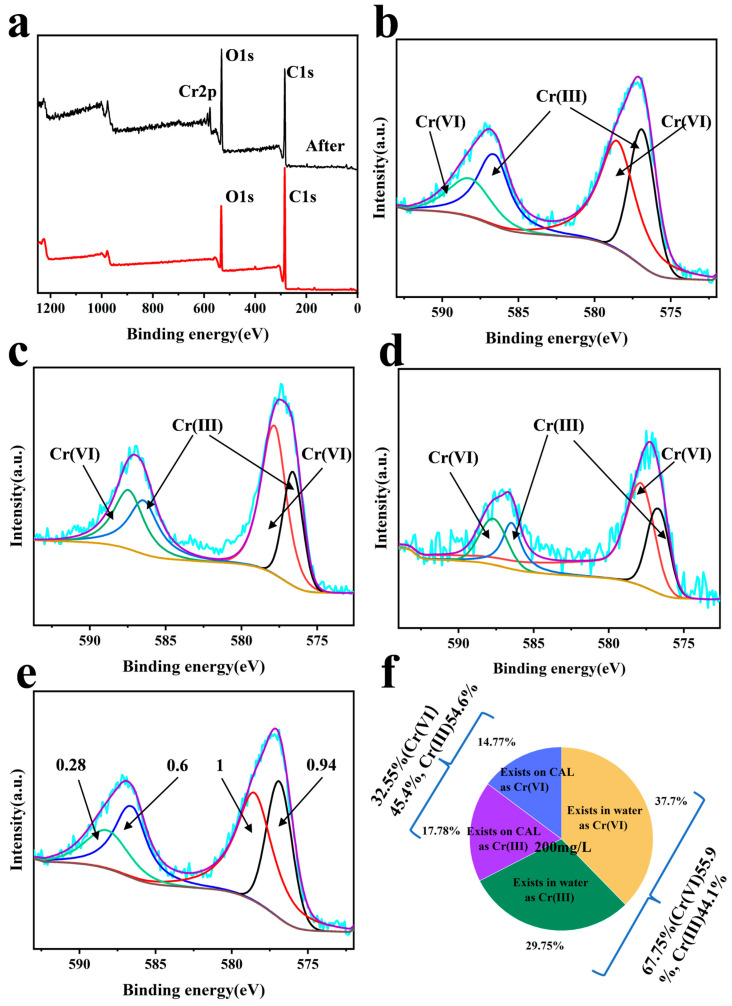
XPS analysis of (**a**) CAL before and after adsorption chromium (pH = 2, C_0_ = 200 mg L^−1^, V = 100 mL, CAL dosage = 25 mg, t = 24 h); (**b**) used CAL in pH = 2; (**c**) used CAL in pH = 5; (**d**) used CAL in pH = 7; (**e**) XPS analysis of ratio of peak areas (C_0_ = 200 mg L^−1^, pH = 2, CAL dosage = 25 mg, t = 24 h); and (**f**) forms of existence of Cr (at%) (C_0_ = 200 mg L^−1^, V = 100 mL, pH = 2, CAL dosage = 25 mg, t = 24 h).

As shown in Figure 6, the Cr2p_3/2_ spectrum shows distinct features—the characteristic peak at 577.5 eV (accounting for 54.6% of total chromium) verifies the formation of Cr (III), while the residual peak at 580.1 eV indicates partial adsorption of Cr (VI) without reduction. Most notably, the appearance of a satellite peak at 586.2 eV provides direct evidence for charge transfer from catechol’s π-electrons to Cr (VI), further substantiating the reduction mechanism. This confirmed the successful adsorption of chromium by CAL. Further analyzing the croup peaks, Cr (VI) and Cr (III) with different proportions appeared following the pH value increases. The percentage of Cr (III) decreased from 54.61% to 40.83% and 39.12% when the pH value of the solution was 2, 5, and 7. The ratio of adsorbed Cr (VI)/Cr (III) was 0.83, 1.45, and 1.56. This may indicate that CAL, Cr (III), and Cr (VI) interact with each other, as shown in Figure 6e, and that other cations can replace CrO_4_^2−^. Based on the percentage of Cr (III) in solution and on CAL, this indicated the importance of H^+^ in the reduction of Cr (VI): the fewer H^+^, the less reduction. According to the area ratio of xps peaks after the adsorption of chromium ions by CAL (Figure 6e) and the changes in the Cr (III) and Cr (VI) components in the solution, as shown in Figure 4a–d of the previous section, a comprehensive analysis was carried out to derive the specific proportions of reduction and adsorption in the purification of Cr (VI) by CAL, as shown in Figure 6f (C_0_ = 200 mg L^−1^, pH = 2, CAL dosage = 25 mg, t = 24 h).

**Figure 7:**
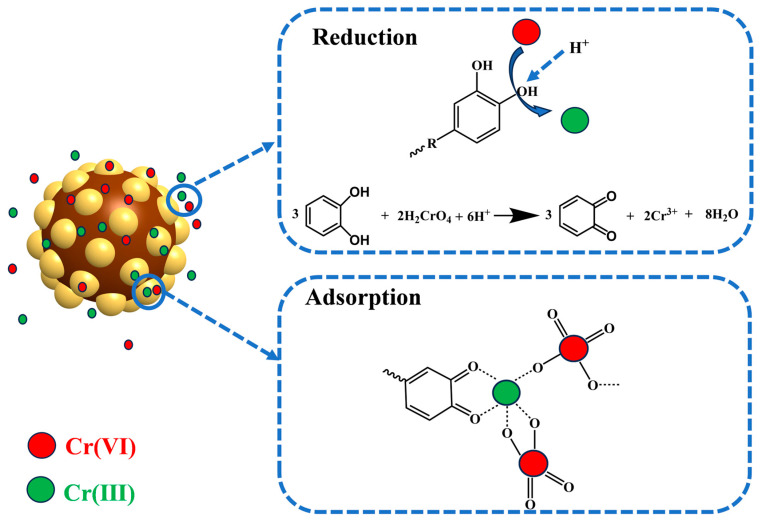
Schematic diagram of the adsorption–reduction procedure of Cr (VI) over CAL.

The FTIR spectral evolution clearly demonstrates the redox transformation: the vanishing 1357 cm^−1^ peak (C-O-H stretching) directly evidences catechol’s oxidation to quinone during Cr (VI) reduction, while the concomitant intensification of the 1506 cm^−1^ band (aromatic C = C vibration) verifies the preservation of π-conjugated structures throughout the modification process. The possible adsorption mechanism is shown in Figure 7.

### 3.7. Comparison of Cr (VI) Absorptivity with Others

Compared with other reported adsorbents (Table 1), the prepared bio-based CAL exceeded most of them in the removal of harmful Cr (VI). This remarkable performance largely stems from the unique reaction mechanism of CAL, which fundamentally differs from conventional adsorbents that only exhibit adsorption capability. Unlike these single-function materials, CAL’s catechol groups enable the simultaneous adsorption and reduction of Cr (VI) to Cr (III) in a single treatment step, thereby eliminating the need for secondary remediation processes. This implies that lignin and its derivatives have potential applications in the field of Cr (VI) adsorption.

### 3.8. Isothermal and Adsorption Kinetic Modeling

The adsorption behavior of CAL was analyzed using four models: the Langmuir isothermal model [54] (Figure S2b), the Freundlich isothermal model [55] (Figure S2a), the pseudo-primary model (Figure S2c), and the pseudo-secondary model (Figure S2d) [56,57]. The ultimate findings indicate that the Langmuir isotherm model and the pseudo-second-order model are apt at describing the adsorption process of CAL on Cr (VI) (Supporting Information).

### 3.9. Effects of Competing Ions

**Figure 8:**
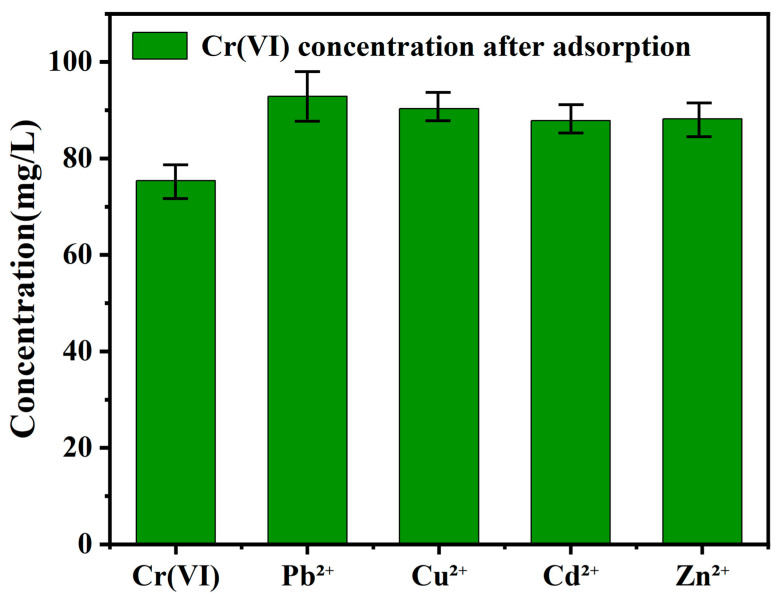
Effects of competing ions on the adsorption of Cr (Ⅵ) by CAL.

Figure 8 demonstrates the competitive adsorption performance of the CAL material for Cr (VI) removal in multicomponent systems containing Pb^2+^, Cu^2+^, Cd^2+^, and Zn^2+^. Cr (VI) maintains significantly higher removal efficiency compared to other metal ions, with only a modest reduction in performance relative to single-component systems, confirming the robustness of its adsorption–reduction mechanism. Among the competing ions, Pb^2^⁺ shows the strongest interference due to its high affinity for phenolic groups, while Cu^2^⁺ exhibits concentration variations suggesting potential redox interactions. The material demonstrates multifunctional removal capability for mixed metal systems while preserving excellent Cr (VI) uptake. These results validate CAL’s effectiveness under realistic wastewater conditions where multiple heavy metal contaminants coexist.

## 4. Conclusions

In conclusion, CAL, a bio-based adsorbent material, was successfully developed by phenolizing alkali lignin with catechol and further cross-linking by acetone. This CAL exhibits outstanding Cr (VI) removal capacity (498.4 mg/g), significantly surpassing most reported bio-based adsorbents, while uniquely combining adsorption with efficient Cr (VI)-to-Cr (III) reduction. This dual functionality stems from catechol’s redox-active phenolic groups, though the system shows pronounced pH dependence, achieving maximum performance at pH 2–4, where protonated catechol groups optimally interact with HCrO_4_^−^ species. While the pH sensitivity currently limits broad application, the use of industrial lignin byproducts provides distinct sustainability advantages. Future development will focus on expanding the operational pH range through lignin source selection and molecular modifications while advancing toward practical implementation through pilot-scale validation.

## Figures and Tables

**Table 1:** Comparison of Cr (VI) absorptivity of CAL with other reported materials.

Adsorbent	pH	Q_m_ (mg g^−1^)	Ref.
LGNs	2.0	368.78	[47]
g-C_3_N_4_	2.0	142.85	[48]
CTS-VAN	3.0	188.68	[49]
ECS	2.0	144.55	[50]
Fe_3_O_4_-SiO_2_-CTS-PEI	2.5	236.4	[51]
DOTA@Sludge@Chitosan	3	273.3	[52]
PTHA	2.5	283.9	[53]
CAL	2.0	498.4	This work

## Data Availability

The original contributions presented in this study are included in the article/Supplementary Material. Further inquiries can be directed to the corresponding authors.

## Supplementary Materials

The following supporting information can be downloaded at: https://www.mdpi.com/article/10.3390/polym17121658/s1.

## References

[1] Koleli N., Demir A., Prasad M.N.V., Shih K. (2016). Chapter 11—Chromite. Environmental Materials and Waste.

[2] du Preez S.P., van Kaam T.P.M., Ringdalen E., Tangstad M., Morita K., Bessarabov D.G., van Zyl P.G., Beukes J.P. (2023). An Overview of Currently Applied Ferrochrome Production Processes and Their Waste Management Practices. Minerals.

[3] Coetzee J.J., Bansal N., Chirwa E.M.N. (2018). Chromium in Environment, Its Toxic Effect from Chromite-Mining and Ferrochrome Industries, and Its Possible Bioremediation. Expo. Health.

[4] Chen J., Zhang X., Wang Y., Tan C., Zhou M., Tian Y., Li L., Qiu F. (2024). Research progress on hazardous chromite ore processing residue treatment and utilization: A critical review. Chem. Eng. Commun..

[5] Miretzky P., Cirelli A.F. (2010). Cr(VI) and Cr(III) removal from aqueous solution by raw and modified lignocellulosic materials: A review. J. Hazard. Mater..

[6] Iyer M., Anand U., Thiruvenkataswamy S., Babu H.W.S., Narayanasamy A., Prajapati V.K., Tiwari C.K., Gopalakrishnan A.V., Bontempi E., Sonne C. (2023). A review of chromium (Cr) epigenetic toxicity and health hazards. Sci. Total Environ..

[7] Khoo P.S., Ilyas R.A., Aiman A., Wei J.S., Yousef A., Anis N., Zuhri M.Y.M., Abral H., Sari N.H., Syafri E. (2024). Revolutionizing wastewater treatment: A review on the role of advanced functional bio-based hydrogels. Int. J. Biol. Macromol..

[8] Xing R.F., Song Y.X., Gao T.T., Cai X.X., Yao J.S., Liu Q.Z., Zhang C.B. (2023). High capacity and fast removal of Cr(vi) by alkali lignin-based poly(tetraethylene pentamine-pyrogallol) sorbent. Rsc Adv..

[9] Sharma P., Singh S.P., Tripathi R.D., Tong Y.W. (2023). Chromium toxicity and tolerance mechanisms in plants through cross-talk of secondary messengers: An overview of pathways and mechanisms. Environ. Pollut..

[10] Yan G., Gao Y., Xue K., Qi Y., Fan Y., Tian X., Wang J., Zhao R., Zhang P., Liu Y. (2023). Toxicity mechanisms and remediation strategies for chromium exposure in the environment. Front. Environ. Sci..

[11] Swaroop A., Bagchi M., Preuss H.G., Zafra-Stone S., Ahmad T., Bagchi D., Vincent J.B. (2019). Chapter 8—Benefits of chromium(III) complexes in animal and human health. The Nutritional Biochemistry of Chromium (III).

[12] Cojocaru C., Clima L. (2020). Polymer assisted ultrafiltration of AO7 anionic dye from aqueous solutions: Experimental design, multivariate optimization, and molecular docking insights. J. Membr. Sci..

[13] Li X., Xiang X.-y., Wu Y.-x., Sun Y.-m., Wei Y.-f. (2024). Separation of V(V) and Cr(VI) from vanadium-containing solutions based on E-pH diagrams of V-Cr-S-H2O system. Sep. Purif. Technol..

[14] Jiang Y., Tian Q., Xu J., Qiu F., Zhang T. (2024). Enhanced separation of dual pollutants from wastewater containing Cr(VI) and oil via Fe-doped sludge derived membrane. Chem. Eng. Sci..

[15] Kohila N., Subramaniam P. (2020). Removal of Cr(VI) using polyaniline based Sn(IV), Ce(IV) and Bi(III) iodomolybdate hybrid ion exchangers: Mechanistic and comparative study. J. Environ. Chem. Eng..

[16] Luo Y., Lan Y., Liu X., Xue M., Zhang L., Yin Z., He X., Li X., Yang J., Hong Z. (2023). Hydrochar effectively removes aqueous Cr(VI) through synergistic adsorption and photoreduction. Sep. Purif. Technol..

[17] Wang H., Wang W., Zhou S., Gao X. (2023). Adsorption mechanism of Cr(VI) on woody-activated carbons. Heliyon.

[18] Tu W., Cai W. (2024). Selective Adsorption of Hazardous Substances from Wastewater by Hierarchical Oxide Composites: A Review. Toxics.

[19] Crini G., Lichtfouse E., Wilson L.D., Morin-Crini N. (2018). Conventional and non-conventional adsorbents for wastewater treatment. Environ. Chem. Lett..

[20] Vara D., Jha S., Bisht S., Shahabuddin S., Gaur R., Suhas, Tyagi I. (2024). Sustainable Bio-Based Adsorbents for Simultaneous and Efficient Removal of Hazardous Dyes from Aqueous Solutions. Toxics.

[21] Kolya H., Kang C.-W. (2023). Bio-Based Polymeric Flocculants and Adsorbents for Wastewater Treatment. Sustainability.

[22] Zhou H.R., Huang J., Chen M., Li Y., Yuan M., Yang H. (2021). Effect of metal ions with reducing properties on hydrogels containing catechol groups. Colloids Surf. A Physicochem. Eng. Asp..

[23] Ko S., Baek M.-J., Wi T.-U., Kim J., Park C., Lim D., Yeom S.J., Bayramova K., Lim H.Y., Kwak S.K. (2022). Understanding the Role of a Water-Soluble Catechol-Functionalized Binder for Silicon Anodes by Diverse In Situ Analyses. ACS Mater. Lett..

[24] Chio C., Sain M., Qin W. (2019). Lignin utilization: A review of lignin depolymerization from various aspects. Renew. Sustain. Energy Rev..

[25] Chen M., Li Y., Liu H., Zhang D., Guo Y., Shi Q.-S., Xie X. (2024). Lignin hydrogenolysis: Tuning the reaction by lignin chemistry. Int. J. Biol. Macromol..

[26] Guo X.-j., Fu W.-k., Ma J.-y., Xi B.-j., Wang C., Guan M.-y. (2024). Efficient removal of Cr(VI) by polydopamine-modified lignin from aqueous solution: Batch and XAFS studies. Water Sci. Eng..

[27] Liu Z.-H., Li B.-Z., Yuan J.S., Yuan Y.-J. (2022). Creative biological lignin conversion routes toward lignin valorization. Trends Biotechnol..

[28] Kocaturk E., Salan T., Ozcelik O., Alma M.H., Candan Z. (2023). Recent Advances in Lignin-Based Biofuel Production. Energies.

[29] Mateo S., Fabbrizi G., Moya A.J. (2025). Lignin from Plant-Based Agro-Industrial Biowastes: From Extraction to Sustainable Applications. Polymers.

[30] Zhao C.K., Li S.X., Zhang H., Yue F.X., Lu F.C. (2020). Structural insights into the alkali lignins involving the formation and transformation of arylglycerols and enol ethers. Int. J. Biol. Macromol..

[31] Yadav P., Kring J., Gogate P.R. (2025). Ultrasound-assisted synthesis of carboxy-methyl lignin from sawdust based lignin as a sustainable source. Chem. Eng. Process.-Process Intensif..

[32] Gong L., Wu H., Shan X., Li Z. (2021). Facile fabrication of phosphorylated alkali lignin microparticles for efficient adsorption of antibiotics and heavy metal ions in water. J. Environ. Chem. Eng..

[33] Li J.-F., Li Z.-M., Xu J.-B., Guo C.-Y., Fang G.-W., Zhou Y., Sun M.-S., Tao D.-J. (2024). Demethylation of Waste Alkali Lignin for Rapid and Efficient Ammonia Adsorption. Ind. Eng. Chem. Res..

[34] Zhang Z., Liu Q., Gao T., Qiao C., Yao J., Zhang C. (2022). Phenolation of lignin for polycatecholamines to remove Cr (VI). J. Water Process Eng..

[35] Hagiri M., Fukuhara S., Kimura Y., Manaka A. (2024). Quantitative determination of Hexavalent chromium using a microtiter plate: Analytical performance, operational efficiency, and fixation of a colorimetric reagent in the plate wells. Microchem. J..

[36] Martins A.S., Dantas H.A., Dantas K.F. (2023). Determination of trace elements in mineral water by MIP OES using the Marin-5 nebulization system. J. Anal. At. Spectrom..

[37] Abdulkhani A., Khorasani Z., Hamzeh Y., Momenbeik F., Zadeh Z.E., Sun F., Madadi M., Zhang X. (2022). Valorization of bagasse alkali lignin to water-soluble derivatives through chemical modification. Biomass Convers. Biorefinery.

[38] Morales A., Labidi J., Gullón P. (2022). Influence of lignin modifications on physically crosslinked lignin hydrogels for drug delivery applications. Sustain. Mater. Technol..

[39] Feng C.-y., Chen H.-j., Yang M.-y., Feng Z.-s., Wang Y. (2022). Metallization of Polyphenylene Sulfide by Low-Cost Mussel-Inspired Catechol/Polyamine Surface Modification. ACS Appl. Polym. Mater..

[40] El Mansouri N.E., Yuan Q.L., Huang F. (2011). Study of chemical modification of alkaline lignin by the glyoxalation reaction. BioResources.

[41] Vallejos M.E., Felissia F.E., Curvelo A.A.S., Zambon M.D., Ramos L., Area M.C. (2011). Chemical and physico-chemical characterization of lignins obtained from ethanol-water fractionation of bagasse. BioResources.

[42] Apaydın Varol E., Mutlu Ü. (2023). TGA-FTIR Analysis of Biomass Samples Based on the Thermal Decomposition Behavior of Hemicellulose, Cellulose, and Lignin. Energies.

[43] Nair V., Panigrahy A., Vinu R. (2014). Development of novel chitosan-lignin composites for adsorption of dyes and metal ions from wastewater. Chem. Eng. J..

[44] Zhang Y., Liu Q., Ma W., Liu H., Zhu J., Wang L., Pei H., Liu Q., Yao J. (2022). Insight into the synergistic adsorption-reduction character of chromium(VI) onto poly(pyrogallol-tetraethylene pentamine) microsphere in synthetic wastewater. J. Colloid Interface Sci..

[45] Xu J., Dai Y., Shi Y., Zhao S., Tian H., Zhu K., Jia H. (2020). Mechanism of Cr(VI) reduction by humin: Role of environmentally persistent free radicals and reactive oxygen species. Sci. Total Environ..

[46] Karimova N.V., Luo M., Sit I., Grassian V.H., Gerber R.B. (2022). Absorption Spectra and the Electronic Structure of Gallic Acid in Water at Different pH: Experimental Data and Theoretical Cluster Models. J. Phys. Chem. A.

[47] Yan Z., Wu T., Fang G., Ran M., Shen K., Liao G. (2021). Self-assembly preparation of lignin–graphene oxide composite nanospheres for highly efficient Cr(vi) removal. RSC Adv..

[48] Anush S.M., Naga Raju S., Gayathri B.H., Ajeya K.P., Girish Y.R., Darshan S., Naveen Y.P., Prashantha K., Narendra B.K., Jayaram A. (2024). Graphitic C3N4 incorporated chitosan-poly(vinyl alcohol) blend nanocomposites for the removal of Cu(II) and Cr(VI) ions from aqueous solutions. Express Polym. Lett..

[49] Khalil T.E., Abdel-Salam A.H., Mohamed L.A., El-Meligy E., El-Dissouky A. (2023). Crosslinked modified chitosan biopolymer for enhanced removal of toxic Cr(VI) from aqueous solution. Int. J. Biol. Macromol..

[50] Zhang S., Xin L., Li M., Fan F., Long H., Gao X. (2023). Synthesis of Amino-protected Chitosan by Tripolyphosphate and Epichlorohydrin Modification: Cr(VI) Adsorption and Reaction Mechanism. J. Polym. Environ..

[51] Sun X., Yang L., Dong T., Liu Z., Liu H. (2015). Removal of Cr(VI) from aqueous solution using amino-modified Fe_3_O_4_–SiO_2_–chitosan magnetic microspheres with high acid resistance and adsorption capacity. J. Appl. Polym. Sci..

[52] Xu K., He T., Li L., Iqbal J., Tong Y., Hua L., Tian Z., Zhao L., Li H. (2024). DOTA functionalized adsorbent DOTA@Sludge@Chitosan derived from recycled shrimp shells and sludge and its application for lead and chromium removal from water. Int. J. Biol. Macromol..

[53] Liu Q., Liu Q., Liu B., Hu T., Liu W., Yao J. (2018). Green synthesis of tannin-hexamethylendiamine based adsorbents for efficient removal of Cr(VI). J. Hazard. Mater..

[54] Kaiblinger N., Hahn R., Beck J., Wang Y., Carta G. (2023). Direct calculation of the equilibrium composition for multi-component Langmuir isotherms in batch adsorption. Adsorption.

[55] Petroli G., Brocardo de Leon V., Di Domenico M., Batista de Souza F., Zanini Brusamarello C. (2024). Application of artificial neural networks and Langmuir and Freundlich isotherm models to the removal of textile dye using biosorbents: A comparative study among methodologies. Can. J. Chem. Eng..

[56] Al-Arjan W.S. (2023). Self-Assembled Nanofibrous Membranes by Electrospinning as Efficient Dye Photocatalysts for Wastewater Treatment. Polymers.

[57] Ali S.I., Lalji S.M., Awan Z., Hashmi S., Khan G., Asad M. (2023). Comprehensive performance analysis of kinetic models used to estimate asphaltene adsorption kinetics on nanoparticles. Chem. Pap..

