# Supplementary material for: Catechol-Modified Alkali Lignin for Cr (VI) Removal from Synthetic Wastewater
Source: Polymers (Basel). 2025 Jun 15;17(12):1658. doi: 10.3390/polym17121658 (PMC12196595; doi:10.3390/polym17121658)
Supplement: Supplementary file 1 [file polymers-17-01658-s001.zip › polymers-3662130-supplementary.pdf]

Catechol-modified alkali lignin for Cr (VI) removal

Supporting Information

1. Isothermal and adsorption kinetic modeling

In order to study the phenomenon of retention, release or migration of solute molecules from the liquid environment to the solid phase when the adsorption process reaches equilibrium at constant temperature and pH. In this study, the concentration at adsorption equilibrium were fitted and analyzed using Langmuir isothermal model (Fig. S. 1 b) and Freundlich isothermal model (Fig. S. 1 a) with the expressions, respectively:

Langmuir isotherm model：

$$\frac{C_e}{Q_e} = \frac{1}{K_L Q_m} + \frac{C_e}{Q_m} \quad (1)$$

Freundlich isotherm model：

$$logQ_e = logK_F + \frac{logC_e}{n} \quad (2)$$

In the equation: C_e is the residual concentration of solute (mg L^{-1}), Q_e is the amount of solute adsorbed by the adsorbent (mg g^{-1}), K_L and K_F are the adsorption equilibrium constants (L mg^{-1}) of Langmuir isothermal model and Freundlich isothermal model, respectively, and n is the constant of the adsorption strength in Freundlich isothermal model.

Among them, the Langmuir isothermal model is based on the assumptions that the adsorption of solutes on the adsorbent is not affected by temperature, the active sites are uniformly distributed on the surface of the adsorbent, and the solutes are adsorbed in a monolayer on the surface of the adsorbent. In contrast, the Freundlich isothermal model assumes that

the distribution of active sites on the adsorbent surface is non-uniform and multilayer adsorption. As shown in Table. S 8, the R^2 (0.98993) of the Freundlich isotherm model is lower than that of the Langmuir isotherm model (0.99305), which suggests that the Langmuir isotherm model is suitable for describing the adsorption process of Cr (VI) by CAL. This also indicates that Cr (VI) adsorbed on the adsorbent adsorbed in monolayers and uniformly adhered to the adsorbent surface. By Langmuir isothermal model, the theoretical Q_m of Cr (VI) adsorbed by CAL at 30 °C was 543.47 mg/g, which was almost the same as that of 498.4 mg/g measured in the experimental process.

Analyzing the adsorption kinetic mechanism is essential for analyzing the adsorption process. The adsorption data at different initial concentrations of Cr (VI) in the batch adsorption experiments were analyzed to obtain the variation curves of the pseudo-primary (Fig. S. 1 c) and pseudo-secondary (Fig. S. 1 d) adsorption kinetic models with the expressions , respectively:

Pseudo first order model:

$$\log(Q_e - Q) = logQ_e - \frac{K_1 t}{2.303} \quad (3)$$

Pseudo second order model:

$$\frac{t}{Q_t} = \frac{1}{K_2 Q_e^2} + \frac{t}{Q_e} \quad (4)$$

where K_1 (h^{-1}) and K_2 (g mg^{-1} h^{-1}) are the pseudo-primary and pseudo-secondary rate constants, and Q_e (mg/g) and Q_t (mg/g) are the adsorption equilibrium adsorption amount and the adsorption amount at any moment,

respectively.

From the data in Table. S 9, it is clear that the parameter R^2 of the pseudo-second-order model (0.99874) is higher than that of the pseudo-first-order model (0.94398), which can more accurately predict the adsorption kinetic behaviors of Cr (VI). The value of Q_e indicates that the theoretical data of the pseudo-second-order equation is consistent with the experimental data. Therefore, the adsorption process follows the pseudo - second-order model, suggesting that the adsorption behavior is achieved through a chemical process of electron sharing or transfer.

In order to have a more in-depth and detailed understanding of the migration and adsorption process of solute molecules in both liquid and solid phases, this paper also analyzes the thermodynamic and kinetic models for the part of the purification process that only plays the role of adsorption, as shown in Figure. S. 2.

From the data in Table. S 10, 11, The adsorption process of chromium ions by CAL is similar to its purification process for hexavalent chromium, both conforming to the Langmuir isothermal model and the pseudo-second-order model. This data again illustrates that the chromium ions adsorbed on the adsorbent adsorbed in monolayers and the adsorption behavior is achieved through a chemical processes of electron sharing or transfer.

Table. S 1 Parameters affecting adsorption by CL, AL and different ratios of the two

Materials	Cr(VI) in solution	Cr(III) in solution	Adsorbed	Initial
CL	0%	100%	0%	200 mg L^{-1}
1:2	75.9%	16.55%	7.5%	200 mg L^{-1}
1:1	56%	32.6%	11.4%	200 mg L^{-1}
2:1	37.7%	29.75%	32.55%	200 mg L^{-1}
3:1	43.75%	28.75%	27.5%	200 mg L^{-1}
4:1	45.65%	30.7%	23.65%	200 mg L^{-1}
AL	76.5%	6.5%	16.55%	200 mg L^{-1}

Conditions ：(pH = 2, C_0 = 200 mg L^{-1}, dosage = 25 mg, V = 100 mL, T = 30 °C, t = 24 h)

Table. S 2 Parameters for different initial concentration affecting adsorption

Conditions	Cr(VI) in solution	Cr(III) in solution	Adsorbed
50 mg L^{-1}	0%	19.8%	80.82%
100 mg L^{-1}	5%	57.95%	37.05%
150 mg L^{-1}	24.8%	39.26%	35.94%
200 mg L^{-1}	37.7%	29.75%	32.55%
250 mg L^{-1}	41.1%	28.4%	30.5%

Conditions ：(pH = 2, dosage = 25 mg, V = 100 mL, T = 30 °C, t = 24 h)

Table. S 3 Parameters for different dosage affecting adsorption

Conditions	Cr(VI) in solution	Cr(III) in solution	Adsorbed	Initial
15 mg	57%	24.66%	18.34%	200 mg L^{-1}
25 mg	37%	29.75%	32.55%	200 mg L^{-1}
35 mg	16.65%	35.85%	47.5%	200 mg L^{-1}
45 mg	3%	41.625%	55.375%	200 mg L^{-1}
55 mg	0%	38.3%	61.7%	200 mg L^{-1}

Conditions ：(pH = 2, C_0 = 200 mg L^{-1}, V = 100 mL, T = 30 °C, t = 24 h)

Table. S 4 Parameters for different pH affecting adsorption

Conditions	Cr(VI) in solution	Cr(III) in solution	Adsorbed	Initial
2	37.7%	29.75%	32.55%	200 mg L^{-1}
3	56.25%	11.25%	32.5%	200 mg L^{-1}
4	75.45%	1.425%	23.375%	200 mg L^{-1}
5	78.95%	1.55%	19.5%	200 mg L^{-1}
6	79.6%	1.4%	19%	200 mg L^{-1}
7	80.15%	2.85%	17%	200 mg L^{-1}

Conditions ：(C_0 = 200 mg L^{-1}, dosage = 25 mg, V = 100 mL, T = 30 °C, t = 24 h)

Table. S 5 Parameters for different temperature affecting adsorption

Conditions	Cr(VI) in solution	Cr(III) in solution	Adsorbed	Initial
20 ℃	43.75%	26.75%	29.5%	200 mg L^{-1}
30 ℃	37.7%	29.75%	32.55%	200 mg L^{-1}
40 ℃	35%	28.75%	36.25%	200 mg L^{-1}

Conditions ：(pH = 2, C_0 = 200 mg L^{-1}, dosage = 25 mg, V = 100 mL, t = 24 h)

Table. S 6 Parameters for different time affecting adsorption

Conditions	Cr(VI) in solution	Cr(III) in solution	Adsorbed	Initial
0 h	100%	0%	0%	200 mg L^{-1}
0.5 h	64.75%	18.9%	16.35%	200 mg L^{-1}
2 h	51.4%	29.7%	18.9%	200 mg L^{-1}
4 h	50.3%	30.05%	19.65%	200 mg L^{-1}
8 h	42.6%	34.8%	22.6%	200 mg L^{-1}
12 h	39.7%	36.55%	23.75%	200 mg L^{-1}
24 h	37.7%	29.75%	32.55%	200 mg L^{-1}

Conditions ：(pH = 2, C_0 = 200 mg L^{-1}, dosage = 25 mg, V = 100 mL, T = 30 °C)

Table. S 7 Parameters for different time affecting adsorption

Conditions	Cr(VI) in solution	Cr(III) in solution	Adsorbed	Initial
0 h	100%	0%	0%	50 mg L^{-1}
0.5 h	48.8%	13.12%	38.12%	50 mg L^{-1}
2 h	20.4%	23.42%	56.18%	50 mg L^{-1}
4 h	7.5%	22.82%	68.18%	50 mg L^{-1}
8 h	0.75%	28.03%	71.22%	50 mg L^{-1}
12 h	0%	19.78%	80.22%	50 mg L^{-1}
24 h	0%	19.78%	80.22%	50 mg L^{-1}

Conditions ：(pH = 2, C_0 = 50 mg L^{-1}, dosage = 25 mg, V = 100 mL, T = 30 °C)

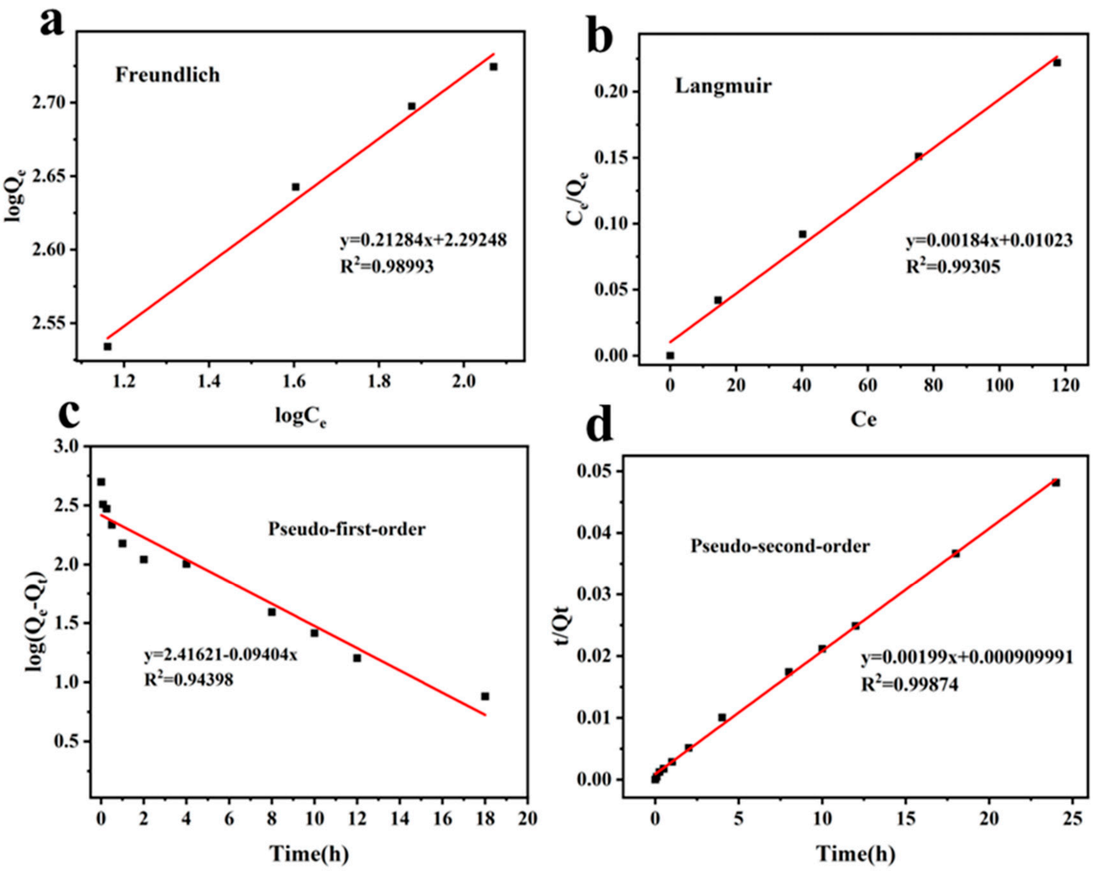

Figure. S. 1. Linear fittings of (a) Langmuir and (b) Freundlich isotherm models, (c) the pseudo-first-order and (d) pseudo-second-order equations for removal of Cr (VI) by CAL.

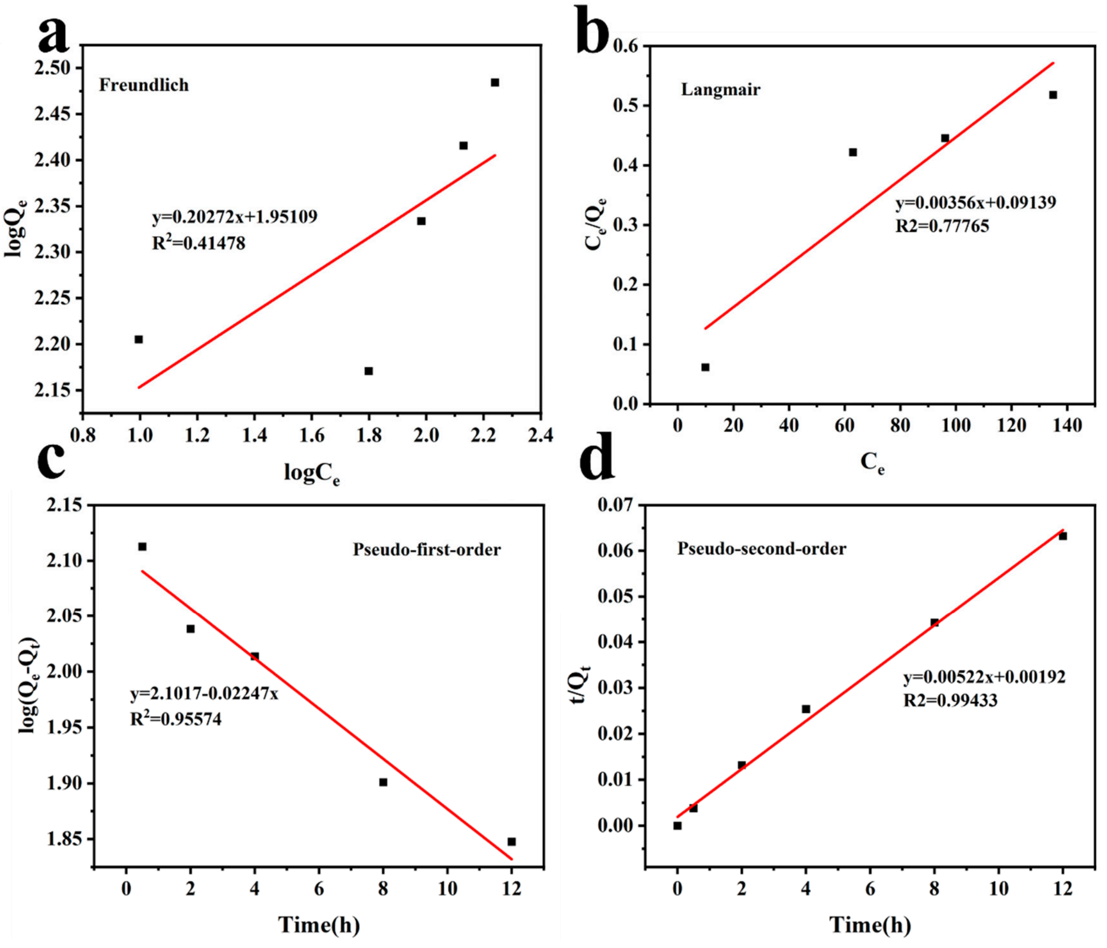

Figure. S. 2. Linear fittings of (a) Langmuir and (b) Freundlich isotherm models, (c) the pseudo-first-order and (d) pseudo-second-order equations for adsorption of Cr by CAL.

Table. S 8 Fitting parameters of isothermal adsorption model at different temperatures

Models	Langmuir isotherm model			Freundlich isotherm model		
Parameters	K_L (L/mg)	Q_m (mg/g)	R^2	K_F (mg/g)/(mg/L)1/n	1/n	R^2
Cr (VI)	0.1799	543.47	0.99305	196.101	0.2128	0.98993

Table. S 9 Quasi-primary and secondary kinetic parameters of Cr(VI) adsorption by CAL

Models	Pseudo first order kinetic			Pseudo second order kinetic		
Parameters	K_1 (1/h)	Q_e(mg/g)	R^2	K_2(g/mg h)	Qe(mg/g)	R^2
Cr(VI)	0.2166	260.7414	0.94398	0.004352	502.5126	0.99874

Table. S 10 Fitting parameters of isothermal adsorption model at different temperatures

Models	Langmuir isotherm model			Freundlich isotherm model		
Parameters	K_L (L/mg)	Q_m (mg/g)	R^2	K_F (mg/g)/(mg/L)1/n	1/n	R^2
Cr (VI)	0.03895	280.9	0.77765	89.349	0.20272	0.41478

Table. S 11 Quasi-primary and secondary kinetic parameters of Cr adsorption by CAL

Models	Pseudo first order kinetic			Pseudo second order kinetic		
Parameters	K_1 (1/h)	Q_e(mg/g)	R^2	K_2(g/mg h)	Q_e(mg/g)	R^2
Cr(VI)	0.05175	126.3863	0.95574	0.01419	191.5709	0.99433
